# Diffraction-less propagation beyond the sub-wavelength regime: a new type of nanophotonic waveguide

**DOI:** 10.1038/s41598-019-41810-0

**Published:** 2019-03-29

**Authors:** Carlos Alonso-Ramos, Xavier Le Roux, Jianhao Zhang, Daniel Benedikovic, Vladyslav Vakarin, Elena Durán-Valdeiglesias, Dorian Oser, Diego Pérez-Galacho, Florent Mazeas, Laurent Labonté, Sébastien Tanzilli, Éric Cassan, Delphine Marris-Morini, Pavel Cheben, Laurent Vivien

**Affiliations:** 1 Centre for Nanoscience and Nanotechnology, CNRS, Université Paris-Sud, Université Paris-Saclay, C2N – Orsay, 91120 Palaiseau, France; 2 Centre for Optical and Electromagnetic Research, Zijingang Campus, Zhejiang University, Hangzhou, 310058 China; 3 Université Côte d’Azur, CNRS, Institut de Physique de Nice (INPHYNI), Parc Valrose, 06108 Nice Cedex 2, France; 4 National Research Council, Ottawa, K1A 0R6 Canada; 5 Photonics Reseach Labs, iTEAM Research Institute, Universitat Politecnica de Valencia, Valencia, Spain

## Abstract

Sub-wavelength grating (SWG) metamaterials have garnered a great interest for their singular capability to shape the propagation of light. However, practical SWG implementations are limited by fabrication constraints, such as minimum feature size. Here, we present a new nanophotonic waveguide grating concept that exploits phase-matching engineering to suppress diffraction effects for a period three times larger than those with SWG approaches. This long-period grating not only facilitates fabrication, but also enables a new diffraction-less regime with additional degrees of freedom to control light propagation. More specifically, the proposed phase-matching engineering enables selective diffraction suppression, providing new tools to shape propagation in the grating. We harness this flexible diffraction control to yield single-mode propagation in, otherwise, highly multimode waveguides, and to implement Bragg filters that combine highly-diffractive and diffraction-less regions to dramatically increase light rejection. Capitalizing on this new concept, we experimentally demonstrate a Si membrane Bragg filter with record rejection value exceeding 60 dB. These results demonstrate the potential of the proposed long-period grating for the engineering of diffraction in nanophotonic waveguides and pave the way for the development of a new generation of high-performance Si photonics devices.

## Introduction

Silicon photonics is widely recognized as an enabling technology for next generation optical interconnects, holding the promise of providing ultra-compact and low power consumption opto-electronic transceivers, fabricated at large volumes leveraging existing CMOS facilities^1^. Driven by the impressive technological development over the recent years, silicon photonics is expanding its frontiers towards new applications beyond datacom^2^. These include, among others, chemical and biological sensing^3^, radio-over-fiber^4^, spectroscopy^5^, and quantum cryptography^6^. In this very diverse ecosystem, combining demanding optical interconnects with disruptive new applications, sub-wavelength grating (SWG) engineering has gained momentum due to its unmatched flexibility in controlling the propagation of light in nanophotonic devices^7,8^. SWG metamaterial waveguides rely on periodic silicon patterning, with a structural period shorter than half the wavelength, to synthesize refractive index and chromatic dispersion that can, in principle, be engineered at will^9,10^. Unlike photonic crystals that rely on resonant light confinement^11^, SWG waveguides operate well below the bandgap, guiding light by (synthetic) refractive index difference. This way, they provide flexible control over modal confinement, birefringence and dispersion, non-achievable in conventional waveguide arrangements, alongside with low propagation loss and remarkably wide spectral bandwidth^9,10,12,13^. These key advantages allowed the demonstration of several SWG-based devices with state-of-the-art performance^9,10,12–15^. Still, the comparatively short periods required for SWG operation (typically between 200 nm and 300 nm at wavelengths around 1550 nm) restrict the available design space for metamaterial engineering, especially when considering current large-volume fabrication processes like deep-ultraviolet lithography, with minimum feature sizes of 100–150 nm. The sub-wavelength period can be slightly increased by deconfining the optical mode. However, this solution is constrained by trade-offs in leakage loss into the substrate^16^ and increased minimum bending radius^17^.

Radiation loss can also be minimized in long-period gratings by implementing very weak perturbations (differences lower than 0.01 between high and low index materials). Indeed, long-period gratings, with a periodicity larger than the wavelength, are routinely used in optical fibers for sensing applications^18^, and have been demonstrated in low index contrast integrated waveguides^19^. However, this strategy fails when (high index) silicon waveguides and strong perturbations are considered. Indeed, silicon-on-insulator (SOI) mode converters based on long-period gratings require very weak corrugations and few-microns-long devices to minimize radiation loss^20^.

Here, we propose a radically different approach to suppress diffraction effects in long-period gratings with high index contrast. Rather than minimizing the strength of the corrugation, we mitigate scattering loss by judicious design of phase-matching conditions in the grating. This way, the proposed grating concept allows realizing low-loss nanophotonic waveguide gratings with periods three times larger than those obtained with the SWG approach. The longer non-radiative periods help ease the fabrication constraints and widen the duty cycles that can be fabricated for a given minimum feature size. Furthermore, flexible diffraction engineering in long-period gratings releases new degrees of freedom to shape the propagation of light. Specifically, we show that design of the grating radiation provides selective cancellation of high-order modes. Unlike traditional waveguide geometries, this new approach allows effective single-mode behavior for high-index-contrast fully etched waveguides with micrometric core sizes, opening a new design space. As an example, we further exploit this unique diffraction control to develop a new kind of high-performance Bragg grating filters. The combination of highly-diffractive and diffraction-less regimes provides a dramatic rejection increase, overcoming the rejection-bandwidth trade-off in conventional designs. Based on this concept, we experimentally demonstrate a notch filter with 5 nm bandwidth and rejection depth exceeding 60 dB, among the highest values hitherto reported for Si Bragg filters^21^.

## Results

### Long-period diffraction-less grating

**Figure 1:**
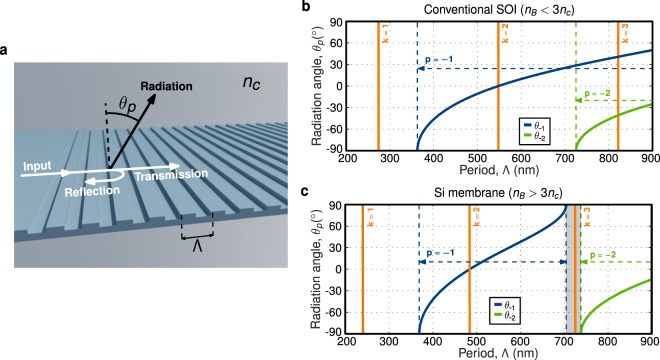
(**a**) Schematic view of a generic waveguide grating, defining input, reflected, transmitted and radiated beams. Reflection and radiation phase-matching conditions in Eqs (1) and (2), respectively, are evaluated as a function of the period (Λ) for TE polarization and wavelength of *λ* = 1550 nm, considering a waveguide grating implemented in (**b**) conventional 220-nm-thick SOI (*n*_*B*_ = 2.83 and *n*_*c*_ = 1.444), and (**c**) 500-nm-thick Si membrane (*n*_*B*_ = 3.2 and *n*_*c*_ = 1). Only in the Si membrane case, fulfilling diffraction-less condition in Eq. (3), a long-period range exists where no radiation orders are allowed (shadowed region between 700 nm and 740 nm).

Conventional waveguide gratings (see example in Fig. 1a) operate in three different regimes^10^: (i) radiation, where the light is scattered away from the grating through excitation of different radiation orders, (ii) reflection, when light is back-reflected by constructive interference of partial reflections (Bragg condition), and (iii) sub-wavelength transmission, where diffraction effects are suppressed, and the grating behaves as a synthetic homogeneous medium. We theoretically prove that, in opposition to the common wisdom, waveguide gratings can operate in transmission mode for periods larger than the propagating wavelength.

Propagation in a waveguide grating is primarily governed by the ratio between the period and the wavelength, through the phase-matching conditions for Bragg reflection^22^ and radiation^23^. Bragg phase-matching condition reads

1 $${\rm{\Lambda }}=\frac{k\lambda }{2{n}_{B}},$$

while radiation condition requires

2 $$|\sin ({\theta }_{p})|=|\frac{{n}_{B}}{{n}_{c}}+\frac{p\lambda }{{n}_{c}{\rm{\Lambda }}}|\le 1.$$

Here *λ* is the wavelength, Λ the pitch, *n*_*B*_ the effective index of the Bloch-Floquet mode in the periodic waveguide, *θ*_*p*_ the radiation angle and *n*_*c*_ the refractive index of the waveguide cladding. Due to the periodicity of the grating, these phase-matching conditions can be satisfied for different reflection and radiation orders, which are accounted by *k* (natural number) for Bragg reflections and *p* (integer number) for radiation. If phase-matching condition in Eq. (1) is fulfilled light is back-reflected. If Eq. (2) is satisfied, light is radiated. Otherwise, light is (ideally) transmitted through the grating with negligible loss.

Sub-wavelength grating waveguides rely on periods below the first order Bragg resonance, Λ < *λ*/(2*n*_*B*_), to suppress diffraction effects^9,10^. In this short-period region no radiation order meets the phase-matching condition in Eq. (2), achieving diffraction-less (and loss-less) propagation through the grating. However, from Eq. (2) it follows that, if the diffraction-less condition

3 $${n}_{B} > 3{n}_{c}$$

is satisfied, a region exists for:

4 $$\lambda /({n}_{B}-{n}_{c}) < {\rm{\Lambda }} < 2\lambda /({n}_{B}+{n}_{c}),$$

where no radiation orders are allowed (see Methods). This is the fundamental principle exploited in this work. It implies that radiation effects are suppressed in high-index-contrast gratings, fulfilling Eq. (3), for a period approximately 3 times larger than the conventional sub-wavelength grating design. The width of this diffraction-less region scales with the factor $({n}_{B}-3{n}_{c})/({n}_{B}^{2}-{n}_{c}^{2})$.

To illustrate the effect of the diffraction-less condition, the performance of waveguide gratings implemented in 500-nm-thick silicon membrane (*n*_*B*_ > 3*n*_*c*_) and conventional 220-nm-thick SOI (*n*_*B*_ < 3*n*_*c*_) are compared. We set a wavelength of 1550 nm and transverse-electric (TE) polarization. For the SOI grating we considered silica cladding (*n*_*c*_ = 1.444) and Bloch-Floquet mode index of *n*_*B*_ = 2.83. In the silicon membrane case, we assumed air cladding (*n*_*c*_ = 1) and *n*_*B*_ = 3.2. Phase-matching conditions in Eqs (1) and (2) are evaluated as a function of the period for gratings implemented in SOI (Fig. 1b) and Si membrane (Fig. 1c). Grating radiation occurs when Eq. (2) is satisfied, i.e. when the radiation angle (blue and green lines) has a real value between −90° and 90°. Vertical orange lines indicate when the Bragg condition (Eq. (1)) is fulfilled, i.e. when light propagated along the periodic structure is reflected. In the case of the conventional SOI grating at least one diffraction order satisfies radiation condition for periods beyond 360 nm. Conversely, in the Si membrane case, radiation and reflection regions are shifted, giving rise to a diffraction-less region for periods between 700 nm and 740 nm where no diffraction order is allowed (shadowed region in Fig. 1c). This diffraction-less region can be exploited to realize long-period nanophotonic waveguide gratings, with relaxed fabrication constraints. The third-order Bragg resonance lies within the diffraction-less region (vertical orange line for *k* = 3 in Fig. 1c), opening a new route to implement high-performance waveguide Bragg filters. Conventional high-order Bragg filters exhibit narrower notch and substantially longer periods, compared to first-order counterparts, but are hampered by radiation loss in the pass band^24^. On the contrary, the proposed grating allows operating around the third-order Bragg resonance without necessarily meeting the radiation condition, overcoming the off-band loss limitation.

### Silicon membrane grating within the diffraction-less regime

To demonstrate the long-period diffraction-less operation we chose to implement a silicon membrane waveguide grating. The extraordinary index contrast between Si and air in membrane waveguides eases satisfying the diffraction-less condition (*n*_*B*_ > 3*n*_*c*_). On the other hand, Si membrane waveguides provide tight mode confinement^25^ and wide transparency^26,27^ that make them a very promising solution for nonlinear applications.

**Figure 2:**
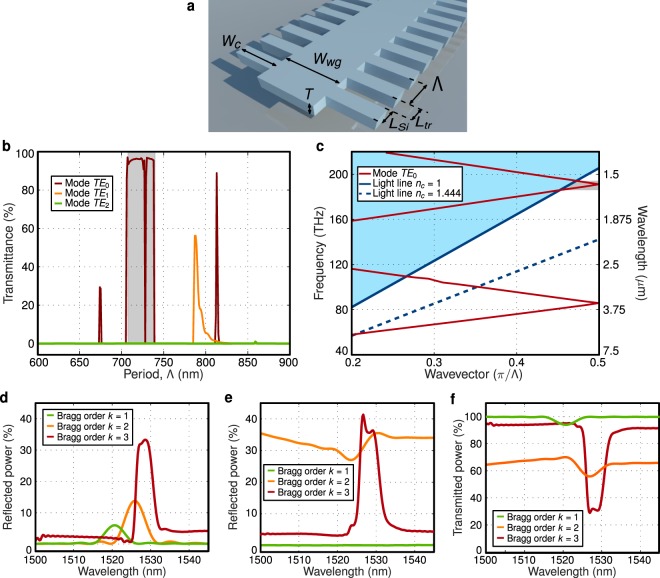
(**a**) Schematic view of the proposed diffraction-less Si membrane grating waveguide with long grating period and waveguide width larger than 1 *μ*m. (**b**) Calculated transmittance for the for the first three modes of the proposed Si membrane waveguide as a function of the period, considering 1-cm-long grating. Diffraction-less light propagation is observed only for the fundamental mode (${n}_{{B}_{0}}=3.2 > 3{n}_{c}$), within the region predicted by Eq. (4). (**c**) Dispersion curve of proposed waveguide grating membrane calculated by 3D FDTD simulations. Light-blue region indicates frequencies above the light line for *n*_*c*_ = 1, i.e. where modes are radiated. Diffraction-less propagation, gray shaded region near 1550 nm wavelength, is observed only when considering the light line with *n*_*c*_ = 1, i.e. when Eq. (3) is satisfied. 3D-FDTD calculations of (**d**) reflectivity, (**e**) radiation, and (**f**) transmission for 50-*μ*m-long Si membrane grating for three different periods, at the first (*k* = 1 and Λ = 240 nm), second (*k* = 2 and Λ = 475 nm) and third (*k* = 3 and Λ = 725 nm) order Bragg resonance conditions.

As schematically shown in Fig. 2a, the proposed device relies in a lateral corrugation, enabling single-etch step definition of the waveguide and the grating. We considered TE polarization and operation near 1550 nm wavelength. The periodic waveguide has a thickness of 500 nm, a width of *W*_*wg*_ = 1.1 *μ*m and a lateral corrugation width of *W*_*c*_ = 500 nm. The grating teeth and trench lengths are *L*_*Si*_ and *L*_*tr*_, respectively, being the period of the structure Λ = *L*_*Si*_ + *L*_*tr*_. A duty cycle of *DC* = *L*_*Si*_/Λ = 0.3 has been considered to promote the third order Bragg resonance^24^.

The comparatively large waveguide transversal dimensions considered here (*W*_*wg*_ = 1.1 *μ*m width by 500 nm thickness) would typically result in a highly-multimode behavior. However, the proposed grating concept allows engineering the geometry of a multi-mode waveguide to make only the fundamental mode (with the highest effective index) satisfy Eq. (3), for which diffraction can be suppressed. Hence, high-order modes will be radiated out by the periodic structure, thereby yielding an effectively single-mode behavior. Specifically, the grating supports three Bloch-Floquet modes, with indices of ${n}_{{B}_{0}}=3.2$, ${n}_{{B}_{1}}=2.9$ and ${n}_{{B}_{2}}=2.5$. Given the air cladding (*n*_*c*_ = 1), only the fundamental mode satisfies the diffraction-less condition. We studied light propagation in this corrugated waveguide using a 2-D Fourier expansion simulation tool with Bloch-Floquet formalism^28^. Figure 2b shows the calculated transmittance for the first three modes in a 1-cm-long grating. The higher order modes, not satisfying the diffraction-less condition, are radiated out. Conversely, the fundamental mode exhibits diffraction-less propagation within the region predicted by the proposed Eq. (3), effectively achieving a single-mode behavior. The diffraction-less region comprises a low-transmittance notch, corresponding to the third-order Bragg resonance for Λ = 3*λ*/(2*n*_*B*_). Here, we exploit this unconventional behavior to demonstrate third-order Bragg Si membrane filters with high-rejection.

The grating structure can also be understood from the point of view of photonic crystals. Modes of a photonic crystal are typically characterized by their dispersion curves, compared to the light line^11^. This way, for a given wavevector, modes with a frequency below the light line are guided while modes with a frequency above the light line are radiated. This radiation condition, *f*_*mode*_ ≥ *f*_*lightline*_, is a particular form of the phase-matching condition in Eq. (2). Then, by satisfying the diffraction-less conditions in Eqs (3) and (4), we make sure that the frequency of the mode lies below the light line, thereby avoiding radiation. To illustrate this behavior, Fig. 2c shows the dispersion curves for the fundamental TE mode (*TE*_0_), calculated by 3-D finite difference time domain (FDTD) simulations (with Lumerical Solutions tools), considering a waveguide thickness of 500 nm, a width of 1.1 *μ*m, a corrugation depth of 500 nm and a period of 725 nm. As an example, we plot the light lines for air (*n*_*c*_ = 1) and silica (*n*_*c*_ = 1.444) cladding. Light-blue region indicates frequencies above the light line for *n*_*c*_ = 1, i.e. the region where modes are radiated. Only for *n*_*c*_ = 1 (*n*_*B*_ > 3*n*_*c*_), a diffraction-less region exists for wavelengths near 1550 nm wavelength where the mode frequencies lie below the light line (gray shadowed region in Fig. 2c). This photonic crystal analysis is in good agreement with the previous discussion, describing the diffraction-less design from the point of view of gratings.

To evaluate the performance of the Si membrane gratings as Bragg filters, 3-D FDTD simulations have been performed. We adjust the grating period to yield first (*k* = 1, Λ = 240 nm), second (*k* = 2, Λ = 475 nm) and third (*k* = 3, Λ = 725 nm) order Bragg resonances near 1550 nm wavelength. The reflectivity (Fig. 2d), radiation (Fig. 2e) and transmission (Fig. 2f) for the three Bragg orders are calculated for a grating length of 50 *μ*m. As shown in Fig. 2d, the third order Bragg filter provides the highest reflectivity, exceeding 30%. On the other hand, the three filter orders exhibit very different behavior in terms of radiation. The period for the first order Bragg is below the radiation region, which results in negligible radiation. In contrast, the pitch for second order Bragg fulfills radiation phase-matching condition, Eq. (2), yielding a radiation exceeding 25% in the entire bandwidth. For the third order Bragg, within the diffraction-less region, strong radiation occurs only within the Bragg reflection bandwidth. This results in an atypical and beneficial behavior where both back-reflections and radiation contribute to the high rejection of the filter. From the transmittance calculations shown in Fig. 2b, the slight off-band radiation and reflection for the third order Bragg can be attributed to the mismatch between the non-corrugated waveguide used for excitation and the waveguide grating.

The long-period grating gives access to a third order Bragg resonance surrounded by two diffraction-less regions, at shorter and longer wavelengths, that can be exploited to simultaneously yield narrowband filtering and strong light rejection. Hence, this filter has a great potential to overcome the bandwidth-rejection trade-off in conventional Bragg gratings. First order Bragg filters need to have weak perturbations to yield narrowband operation^29^. On the other hand, higher order Braggs exhibit narrower resonances, but are limited by off-band radiation losses^24^. In the proposed filter, the narrow bandwidth is provided by working with a third-order Bragg resonance and a highly confined mode. In addition, diffraction-less propagation provides low-loss off-band transmission, while the strong rejection is achieved by the combination of back reflections and radiation within the Bragg resonance.

**Figure 3:**
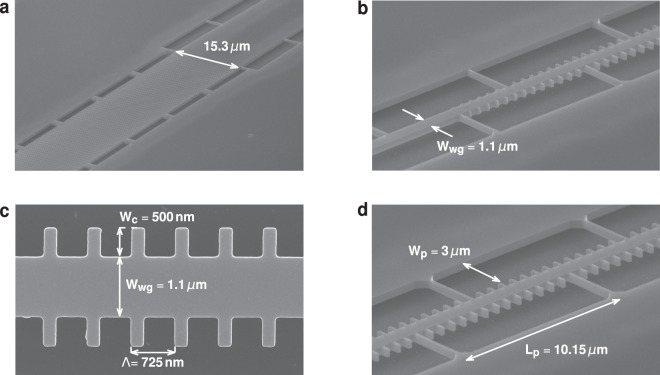
Scanning electron microscope (SEM) image of the fabricated silicon membrane Bragg filter (**a**) fiber-chip grating coupler, (**b**) transition between strip waveguide and grating, (**c**) detail of the filter geometry and (**d**) side view showing anchoring pillars.

We fabricated the suspended Si filters in the SOI platform with 500-nm-thick Si layer and 2-*μ*m-thick buried oxide (BOX) layer. As shown in the scanning electron microscope (SEM) image in Fig. 3, we used a series of equidistantly spaced pillars to anchor the corrugated waveguide to the lateral Si slabs. A pillar width of *W*_*p*_ = 3.5 *μ*m was used to ensure a good optical isolation between the waveguide mode and the lateral silicon slab regions. The separation between pillars of *L*_*p*_ = 10.15 *μ*m (14×Λ) ensures sufficient mechanical stability, while minimizing perturbation of the filter response. Input and output sub-wavelength grating couplers^30^ have been optimized to minimize Fabry-Perot ripples that could distort measured spectral response. Grating couplers are connected to the filters by a short multi-mode strip section and an adiabatic taper.

**Figure 4:**
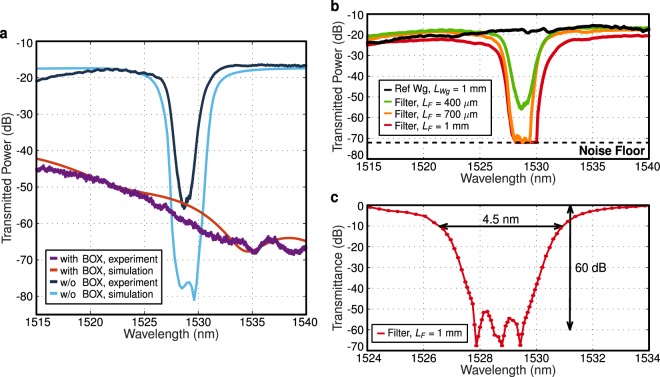
(**a**) 3D-FDTD simulations and experimental transmission spectra of 400 *μ*m-long third-order Bragg grating, with *SiO*_2_ (with BOX) and air under-cladding (w/o BOX). Diffraction-less condition in Eq. (3) is satisfied only for the case with air under-cladding, Si membrane configuration, yielding a 20 dB transmission increase. An offset of −17 dB is added to the 3D-FDTD simulations to account for experimental fiber-chip coupling loss. (**b**) Measured transmitted spectra for third-order Bragg Si membrane filters (Λ = 725 nm) with different lengths, compared with reference (uncorrugated) Si membrane waveguide. (**c**) Transmittance for 1-mm-long filter, measured with high-sensitivity photodetector. In all cases the filters have 500-nm-thick Si, Λ = 725 nm, *W*_*wg*_ = 1.1 *μ*m, *DC* = *L*_*Si*_/Λ = 0.3, *W*_*c*_ = 500 nm, and air top-cladding.

In Fig. 4a we plot the measured transmittance for the fundamental mode of the waveguide grating with a period of 725 nm (third order Bragg resonance *k* = 3) and a length of 400 *μ*m before and after BOX removal with hydrofluoric acid vapor (see Methods). Before the BOX removal the under-cladding index is *n*_*c*_ = 1.444. Hence, the grating does not fulfill diffraction-less condition in Eq. (3), as ${n}_{B} \sim 3.2 < 3{n}_{c}$. This results in strong radiation loss into the substrate. By removing the BOX layer underneath the waveguides, diffraction-less condition is satisfied (*n*_*c*_ = 1 and *n*_*B*_ > 3*n*_*c*_), yielding a remarkable 20 dB improvement in the transmission. These experimental results prove the ability of the proposed phase-matching engineering to substantially reduce radiation loss in long-period gratings. As shown in Fig. 4a, experimental results are in very good agreement with the 3D FDTD simulations. The weaker Bragg rejection in the experiments can be attributed to relative phase mismatch arising from fabrication imperfections.

Figure 4b shows the measured spectra of the fundamental mode of the third-order Si membrane Bragg filters (*k* = 3) with different lengths varying between 400 *μ*m and 1 mm, compared with a reference (non-corrugated) waveguide of 1 mm length. Our grating shows a comparatively good transmission level within the diffraction-less regime, at the two sides of the Bragg resonance. The difference in transmission between the structures can mainly be attributed to the combined effect of sidewall roughness and stitching errors in the electron beam lithography. The filters yield a 3 dB bandwidth of 7 nm and a 10 dB bandwidth of less than 5 nm for a corrugation depth of *W*_*c*_ = 500 nm. For comparison, conventional nanometric-thick SOI Bragg filters require corrugation depths of only 50 nm to achieve similar bandwidth^29^. Despite the presence of fabrication imperfections, our filters yield outstanding rejection levels, with notch transmission below the noise level of the component tester (CT400 Yenista). Note that input and output strip waveguides are multi-mode. Hence, this high rejection stands as a clear evidence of effective single-mode behavior of the waveguide grating. Higher order modes have smaller effective indices, resulting in shorter Bragg resonance wavelengths. Thus, any power transmitted by higher order modes would limit the measured rejection level. The radiation loss of higher order waveguide modes may be accurately measured by implementing mode converters and demultiplexers on chip^31^. However, this study is outside of the scope of this work that targets the demonstration of high-rejection filters with effective single-mode behavior exploiting the proposed diffraction-less regime.

We have further characterized the 1-mm-long filter with a high sensitivity photodetector (OSA Anritsu MS9710B). As shown in Fig. 4c, our filter exhibits a remarkably large rejection, exceeding 60 dB. These results compare to the largest rejections reported for Si Bragg filters^21^, yet relying on a single-etch process and substantially larger period and corrugation widths.

## Discussion

In summary, we have established a new paradigm for the realization of nanophotonic waveguide gratings. Conventional waveguide gratings present three different modes of operation^10^: radiation, Bragg reflection and sub-wavelength transmission. Here we show that a fourth operating mode exists for periods substantially longer than the propagating wavelength, allowing diffraction-less propagation. State-of-the-art sub-wavelength grating waveguides rely on periods shorter than half of the wavelength to suppress diffraction effects^9,10^. On the other hand, detrimental scattering loss has been partially mitigated in long-period gratings by implementing very weak corrugations^20,24^. We propose a radically different approach to suppress diffraction effects in strongly-corrugated long-period gratings, based on the engineering of the phase-matching conditions. Generic design rules are given to yield diffraction-less propagation for a period three times larger than that of standard SWG metamaterial waveguides, substantially relaxing fabrication constraints. Furthermore, this diffraction cancellation strategy unlocks new tools to shape light propagation in nanophotonic waveguides. We show that engineering of diffraction conditions in the proposed grating allows effective single-mode propagation in, otherwise, highly multimode waveguides. The grating geometry can be designed to make only the fundamental mode satisfy the diffraction-less conditions, radiating higher orders out. This effective single-mode behavior opens a new space for the implementation of highly-confined nanophotonic waveguides. We further leverage the unique diffraction control in long-period gratings to develop a new kind of Bragg filters that combine highly diffractive and diffraction-less regions to dramatically increase filter rejection. Both, radiation and back-reflections contribute to reject light within the notch band, while diffraction-less propagation minimizes off-band losses.

Capitalizing on these concepts, we have demonstrated diffraction-less long-period Si membrane gratings, achieving single mode behavior at 1550 nm wavelength for fully-etched waveguide with cross section as large as 1.1 *μ*m wide by 500 nm thick. Such membrane gratings allowed the implementation of third-order Bragg filters with experimental rejection exceeding 60 dB, among the largest values reported for Si Bragg filters^21^. The filter has a 10 dB bandwidth of less than 5 nm for a corrugation depth of *W*_*c*_ = 500 nm. This is almost a ten-fold increase in corrugation depth compared to conventional SOI Bragg filters with similar bandwidth^29^. The third-order Bragg resonance and highly confined mode provide narrow bandwidth, while the combination of back reflections and radiation within the Bragg resonance yield the strong rejection. These results demonstrate the ability of the proposed Bragg grating to overcome the bandwidth-rejection trade-off in conventional filters. More generally, this is the first practical proof of the great potential offered by the diffraction-less regime to shape light propagation in nanophotonic waveguides. We foresee that the diffraction-less approach will expedite the development of a new generation of high-performance nanophotonic circuits exploiting flexible diffraction control in long-period waveguide gratings.

## Methods

### Device fabrication and experimental characterization

For the fabrication of the Si membrane filters, we used electron beam lithography (Nanobeam NB-4 system, 80 kV) and dry etching with an inductively coupled plasma etching (SF_6_/C_4_F_8_) for structure definition. BOX layer was removed to form the suspended structure by exposure to hydrofluoric acid vapor (~1 hour).

For the characterization of the samples we used cleaved SMF-28 fibers at the input and output. A polarization controller was used to set TE polarization at the input grating. A Glan-Thomson polarizer is used at the output, before the photodetector, to remove any residual transverse-magnetic (TM) polarization signal. Fine wavelength scans in Fig. 4a,b, were performed with automatic component tester (CT400 Yenista, noise floor level at around −70 dBm), using a resolution of 10 pm. Filter rejection characterization in Fig. 4c was performed with the high sensitivity photodetector of the OSA Anritsu MS9710B (noise floor level at around −90 dBm) by manual point-by-point scanning of the input wavelength.

### Long-period diffraction-less condition

From Eq. (2), it can be concluded that, for a given wavelength and grating configuration (materials and geometry that set *n*_*B*_) the orders that can radiate are determined by the grating period. In fact, the *p*-th order can radiate only if the period, Λ, satisfies the following condition

5 $${{\rm{\Lambda }}}_{min}^{p} > {\rm{\Lambda }} > {{\rm{\Lambda }}}_{Max}^{p}$$

6 $$\frac{-p\lambda }{{n}_{c}+{n}_{B}} > {\rm{\Lambda }} > \frac{p\lambda }{{n}_{c}-{n}_{B}},$$

where ${{\rm{\Lambda }}}_{min}^{p}$ and ${{\rm{\Lambda }}}_{Max}^{p}$ are the shortest and longest periods allowing radiation in order *p*. In the proposed long-period grating, radiation is precluded by making ${{\rm{\Lambda }}}_{Max}^{-1} < {\rm{\Lambda }} < {{\rm{\Lambda }}}_{min}^{-2}$, i.e. by choosing a period within the range defined in Eq. (4). However, this long-period diffraction-less region exists only if ${{\rm{\Lambda }}}_{Max}^{-1} < {{\rm{\Lambda }}}_{min}^{-2}$. From this requirement it follows that

7 $$\frac{2\lambda }{{n}_{c}+{n}_{B}} < \frac{\lambda }{{n}_{c}-{n}_{B}},$$

which results in the diffraction-less condition in Eq. (3).

## Data Availability

The data that support the findings of this study are available from the corresponding author upon reasonable request.

## References

[1] Vivien, L. & Pavesi, L. Handbook of silicon photonics (CRC Press, 2013).

[2] Thomson D (2016). Roadmap on silicon photonics. J. Opt..

[3] Estevez M-C, Alvarez M, Lechuga LM (2012). Integrated optical devices for lab-on-a-chip biosensing applications. Laser Photonics Rev..

[4] Capmany J, Novak D (2007). Microwave photonics combines two worlds. Nature Photon..

[5] Velasco AV (2013). High-resolution Fourier-transform spectrometer chip with microphotonic silicon spiral waveguides. Opt. Lett..

[6] Silverstone JW (2014). On-chip quantum interference between silicon photon-pair sources. Nat. Photon..

[7] Schmid J (2007). Gradient-index antireflective subwavelength structures for planar waveguide facets. Opt. Lett..

[8] Levy U (2007). Inhomogenous dielectric metamaterials with space-variant polarizability. Phys. Rev. Lett..

[9] Cheben P (2010). Refractive index engineering with subwavelength gratings for efficient microphotonic couplers and planar waveguide multiplexers. Opt. Lett..

[10] Halir R (2015). Waveguide sub-wavelength structures: a review of principles and applications. Laser Photonics Rev..

[11] Joannopoulos, J. D., Johnson, S. G., Winn, J. N. & Meade, R. D. Photonic Crystals: Molding the Flow of Light (Princeton University Press, Princeton, 1995).

[12] Cheben P (2015). Broadband polarization independent nanophotonic coupler for silicon waveguides with ultra-high efficiency. Opt. Express.

[13] Halir R (2016). Ultra-broadband nanophotonic beamsplitter using an anisotropic sub-wavelength metamaterial. Laser Photonics Rev..

[14] Benedikovic D (2016). Single-etch subwavelength engineered fiber-chip grating couplers for 1.3 mm datacom wavelength band. Opt. Express.

[15] Xu Y, Xiao J (2016). Ultracompact and high efficient silicon-based polarization splitter-rotator using a partially-etched subwavelength grating coupler. Sci. Rep..

[16] Sarmiento-Merenguel J (2016). Controlling leakage losses in subwavelength grating silicon metamaterial waveguides. Opt. Lett..

[17] Wang Z, Xu X, Fan D, Wang Y, Chen RT (2016). High quality factor subwavelength grating waveguide micro-ring resonator based on trapezoidal silicon pillars. Opt. Lett..

[18] Bhatia V, Vengsarkar AM (1996). Optical fiber long-period grating sensors. Opt. Lett..

[19] Li L, Burke JJ (1992). Linear propagation characteristics of periodically segmented waveguides. Opt. Lett..

[20] Jin W, Chiang KS (2018). Three-dimensional long-period waveguide gratings for mode-division-multiplexing applications. Opt. Express.

[21] Klitis C, Cantarella G, Strain MJ, Sorel M (2017). High-extinction-ratio TE/TM selective Bragg grating filters on silicon-on-insulator. Opt. Lett..

[22] Pérez-Galacho D (2017). Optical pump-rejection filter based on silicon sub-wavelength engineered photonic structures. Opt. Lett..

[23] Tamir T, Peng S (1977). Analysis and design of grating couplers. Appl. Phys. A-Mater..

[24] Chan SP, Passaro VMN, Mashanovich GZ, Ensell G, Reed GT (2007). Third order Bragg grating filters in small SOI waveguides. J. Europ. Opt. Soc.-Rapid Publications.

[25] McNab SJ, Moll N, Vlasov YA (2003). Ultra-low loss photonic integrated circuit with membrane-type photonic crystal waveguides. Opt. Express.

[26] Wang X, Cheng Z, Xu K, Tsang HK, Xu J-B (2013). High-responsivity graphene/silicon-heterostructure waveguide photodetectors. Nature Photon..

[27] Soler Penadés J (2016). Suspended silicon mid-infrared waveguide devices with subwavelength grating metamaterial cladding. Opt. Express.

[28] Zavargo-Peche L, Ortega-Moñux A, Wangüemert-Pérez JG, Molina-Fernández I (2012). Fourier based combined techniques to design novel sub-wavelength optical integrated devices. Prog. Electromagn. Res..

[29] Wang X, Shi W, Vafaei R, Jaeger NAF, Chrostowski L (2011). Uniform and sampled Bragg gratings in SOI strip waveguides with sidewall corrugations, IEEE Photon. Technol. Lett..

[30] Halir R (2009). Waveguide grating coupler with subwavelength microstructures. Opt. Lett..

[31] Wang J (2015). Broadband and fabrication-tolerant on-chip scalable mode-division multiplexing based on mode-evolution counter-tapered couplers. Opt. Lett..

